# Kansas City Veterinary College

**Published:** 1901-04

**Authors:** 


### KANSAS CITY VETERINARY COLLEGE.

The tenth session of this college, just completed, has been the most successful in its history. The total enrollment of students was fifty-eight; two of this number pursued the post-graduate course on meat-inspection and milk-inspection; thirteen were granted the degree of Doctor of Veterinary Science, ten of whom had completed three full sessions at this college, viz., R. Fred Eagle, Xavie I. Richmond, Charles W. Barnhart, Abram N. Reber, George D. Painter, Arthur Trickett, Arthur W. Miller, Oscar Stuart, John L. Burgett, Hermann J. Timmermann; and three pursuing a post-graduate course leading to the degree as follows: F. F. Brown, D.V.S.; B. F. Kaupp, D.V.S., and C. J. Sibler, V.S.

The commencement exercises were held in the college building on March 14th. The annual address was delivered by Dr. J. L. Short, and a very nice musical programme was furnished by the students.

On Thursday evening, March 7th, the college gave a social dinner to the faculty, students, and friends of the institution at the Midland Hotel, about one hundred covers being laid for the occasion. Dr. S. Stewart, the dean of the faculty, presided as toastmaster, numerous well-chosen subjects being happily responded to. This innovation proved a very agreeable one to students and faculty.

The students of the college maintained an association throughout the session, and its meetings were conducted upon a high plane, affording the participants opportunity to learn methods of conducting associations, and encouraging them to prepare and present papers upon veterinary topics.

